# Identifying the Initiation of a New Line of Therapy for Metastatic Lung, Breast, and Colorectal Cancer in Real‐World Data: A Scoping Review

**DOI:** 10.1002/pds.70370

**Published:** 2026-04-27

**Authors:** Oluwadamilola Onasanya, Seyed Hamidreza Mahmoudpour, Benjamin Bates, Irene M. Shui, Geoffrey Liu, Eng Hooi Tan, Yi‐Hsin Yang, Maribel Salas, Joseph Fadare, Dimitri Bennett, Paula Lana de M. Drummond, Judy Ju‐Young Shin, Sonia Guleria, Jocelyn R. Wang, Manila Hada, Helene Denis, Soko Setoguchi, Ilse Truter, Luciane C. Lopes, Ruth Wangia Dixon

**Affiliations:** ^1^ Carelon Research Wilmington Delaware USA; ^2^ Merck Healthcare KGaA Darmstadt Germany; ^3^ Department of Medicine Rutgers Robert Wood Johnson Medical School New Brunswick New Jersey USA; ^4^ Rutgers Center for Pharmacoepidemiology and Treatment Science (PETS), Rutgers University New Brunswick New Jersey USA; ^5^ Merck & Co. Inc Rahway New Jersey USA; ^6^ University of Toronto Toronto Canada; ^7^ Centre for Statistics in Medicine, Nuffield Department of Orthopaedics, Rheumatology and Musculoskeletal Sciences University of Oxford Oxford UK; ^8^ National Institute of Cancer Research, National Health Research Institutes Tainan Taiwan; ^9^ Bayer Pharmaceuticals Inc Whippany New Jersey USA; ^10^ Center for Real‐World Effectiveness and Safety of Therapeutics (CREST) University of Pennsylvania Perelman School of Medicine Philadelphia Pennsylvania USA; ^11^ Ekiti State University College of Medicine Ado‐Ekiti Nigeria; ^12^ Global Evidence and Outcomes, Takeda Development Center Americas, Inc. Cambridge Massachusetts USA; ^13^ Ezequiel Dias Foundation Belo Horizonte Brazil; ^14^ School of Pharmacy Sungkyunkwan University Seoul South Korea; ^15^ Real World Research (RWD/RWE), Parexel International Stockholm Sweden; ^16^ Janssen R&D Raritan New Jersey USA; ^17^ Merck & Co. Inc Upper Gwynedd Pennsylvania USA; ^18^ Heva Lyon France; ^19^ Drug Utilization Research Unit (DURU) Nelson Mandela University Port Elizabeth (Gqeberha) South Africa; ^20^ Pharmaceutical Science Post‐Graduate Program University of Sorocaba Sorocaba Brazil

**Keywords:** algorithms, frameworks, line of therapy, LOT, observational studies, oncology, scoping review

## Abstract

**Purpose:**

This study aimed to identify and synthesize published algorithms for identifying the initiation of a new line of therapy (LOT) for metastatic lung, breast, and colorectal cancer in real‐world data (RWD).

**Methods:**

We conducted a scoping review of published, English‐language studies describing algorithms for identifying *any LOTs* with systemic anti‐cancer therapy (SACT) for either non‐metastatic or metastatic lung, breast, or colorectal cancer in RWD between January 1, 2014, and April 29, 2024. Dual reviewers independently screened titles, abstracts, and full‐text articles, with disagreements resolved by a third reviewer. Data were extracted, categorized, synthesized, and summarized in narrative and tabular formats.

**Results:**

The review identified 25 studies, mainly (64%) from the United States. Electronic health/medical records (EHRs) were the most frequently utilized (72%) RWD source. Twenty‐four studies (96%) described RWD algorithms for identifying the *initiation of a new LOT for metastatic* lung, breast, or colorectal cancer. In 23 studies, algorithms required observing a new, adjuvant, SACT after an “incident” metastatic diagnosis code, which had been preceded by a metastasis‐free lookback period of varied duration. Three studies' algorithms required observation of the completion of non‐metastatic LOTs before initiation of a new LOT for metastatic cancer. Three studies validated their algorithms.

**Conclusions:**

Different algorithms are being used to identify LOT initiation for metastatic cancer. Most algorithms require an incident diagnosis of metastasis before considering subsequent SACT as newly initiated LOT for metastatic cancer. However, definitions of metastasis onset and gap duration to therapy initiation vary.

## Key Points

- Ninety‐six percent of studies in this review explored ≥ 1 algorithm which defined a new adjuvant SACT observed after a metastasis‐free lookback period and incident diagnosis code for metastatic cancer as new LOT for metastatic cancer.
- However, there was heterogeneity between algorithms in the events signaling metastasis onset, treatment gap durations required before new LOT initiation, and inconsistent querying for completion of non‐metastatic LOTs before identifying new LOT for metastatic cancer.
- Future studies should consider using multiple criteria to ascertain onset of metastatic cancer and query for completion of non‐metastatic LOTs before assessing initiation of the new LOT for metastatic cancer.

## Plain Language Summary

Cancer treatment approaches are evolving. Cancer treatments that affect the whole body (systemic anti‐cancer therapy (SACT)) can now be offered either earlier or later in the treatment plan to both patients with early stages of cancer and those whose cancer has spread. In response to these changes, cancer researchers who analyze routinely collected healthcare data need clear decision rules for differentiating between SACT given for early‐stage cancer (non‐metastatic) and treatments given for cancer that has spread (metastatic). Research has explored different ways to identify when a new line of treatment begins for metastatic lung, breast, or colorectal cancer, using routinely collected healthcare data. Most studies from the past decade agree that evidence of cancer metastasis must be documented in the medical record before labeling subsequent treatments as new lines of metastatic cancer therapy. However, this review highlights differences in the current definitions which researchers working with routine healthcare databases apply to identify patients with metastatic lung, breast, or colorectal cancer and different gap periods being used to distinguish between metastatic, relative to non‐metastatic treatments for cancer. This study also provides a framework for improving the methods for identifying new lines of therapy for metastatic cancer.

## 1 Introduction

Medical treatments for lung, breast, and colorectal cancer are evolving rapidly, requiring updated knowledge and validated algorithms for conducting observational studies on treatment effectiveness, safety, and outcomes [1, 2, 3]. In oncology, a “line of therapy” (LOT) is defined as a serial, chronological number assigned to each systemic anti‐cancer therapy (SACT), representing a distinct attempt to treat a patient with cancer [4, 5]. Each unique LOT is a critical point in a patient's care plan, bearing significant implications for their subsequent treatment and health outcomes (safety, effectiveness, progression‐free disease, and overall survival) [6, 7]. Accurately distinguishing between LOTs in real‐world healthcare databases is essential for researching these outcomes and for harmonizing methods and comparing studies across institutions and geographical regions.

Real‐world data (RWD) is widely used for pharmacoepidemiologic studies of individuals with lung, breast, or colorectal cancer [8, 9, 10]. However, RWD routinely collected from healthcare encounters lacks explicit information to distinguish between curative versus palliative LOTs or between non‐metastatic versus metastatic LOTs. Thus, researchers working with RWD are required to leverage data elements and predefined or newly developed algorithms to distinguish between LOTs for localized/non‐metastatic disease and those for advanced/metastatic disease. However, various approaches are used to distinguish between LOTs in RWD, and treatment practices vary by geographical region.

This scoping review aimed to map and summarize published RWD algorithms for identifying the initiation of LOTs for metastatic lung, breast, or colorectal cancer. The focus was on RWD sources, including electronic health/medical records (EHR), claims, registries, and chart reviews, and on understanding how LOT initiation for metastatic disease is operationalized across geographical regions.

## 2 Methods

A study protocol was registered on Open Science Framework (OSF; link: https://doi.org/10.17605/OSF.IO/TSWY2). This study was conducted in accordance with the Joanna Briggs Institute (JBI) methodology for scoping reviews and followed the Preferred Reporting Items for Systematic Reviews and Meta‐Analyses extension for Scoping Reviews (PRISMA‐ScR) [11, 12].

### 2.1 Eligibility Criteria

Inclusion criteria were: worldwide peer‐reviewed articles or gray literature; published in English language; between 01/01/2014 and 04/29/2024; described any approach for enumerating LOT for any SACT (chemotherapy, endocrine therapy, immunotherapy, or targeted therapy) or combination of SACTs for adults with non‐metastatic or metastatic lung, breast, or colorectal cancer; and the approach was operationalized in RWD (administrative healthcare claims, EHR, cancer registries, or paper medical charts). Studies may have described an approach for identifying either: initiation of LOTs for locally advanced or metastatic disease; or transition point between completion of LOTs for non‐metastatic disease and initiation of LOTs for metastatic disease. Studies describing only approaches for enumerating non‐pharmacological LOT (surgery and radiotherapy) were excluded.

### 2.2 Search Strategy

We implemented a detailed search strategy in MEDLINE (PubMed), Embase, Google Scholar, and the Web of Science Core Collection. Reference lists of all identified studies were hand‐searched for additional references. The search strategy is provided in Supporting Information S1.

### 2.3 Study Selection

**FIGURE 1:**
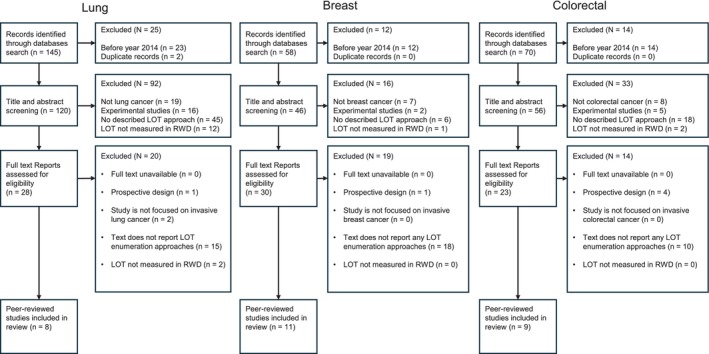
Flow chart for inclusion of study articles. LOT, line of therapy; RWD, real‐world data. Twenty‐five unique studies were included in this scoping review, but there are [3] repetitions of Jeon et al., 2021 and Hess et al., 2021 in more than one cancer type.

Titles and abstracts of all identified citations from our search were uploaded into Smartsheet software (https://app.smartsheet.com/). Two independent reviewers screened titles and abstracts for inclusion. Disagreements were resolved by discussion with a third senior reviewer. Duplicate articles were removed. Potentially relevant studies were retrieved in full text, and their citation details were imported into an abstract repository. The full text of selected articles was thoroughly assessed against the inclusion criteria. We reported reasons for excluding full‐text articles that did not meet the inclusion criteria (Figure 1).

### 2.4 Data Extraction

**FIGURE 2:**
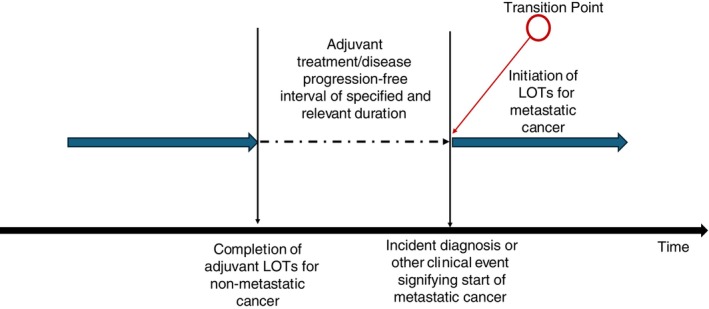
Framework to optimize identification of the transition from lines of therapy for non‐metastatic, to metastatic colorectal cancer in real‐world databases. LOT, line of therapy.

We developed a data extraction tool, adapted from the JBI methodology for scoping reviews, to extract key study findings as outlined in the study protocol (Table S1) [11]. In brief, extracted data included country/region, year, publication type, assessed RWD types, and study population for each article. It also included a query for descriptions of approaches for identifying either the initiation of LOT for locally advanced or metastatic disease or the transition point between completion of LOTs for non‐metastatic disease and initiation of LOT for metastatic disease (Figure 2). Modifications were made to the a priori data extraction tool as published in the protocol (Table S2). Pairs of independent reviewers, organized by cancer type, extracted data from the selected articles into the data extraction tool. Disagreements were resolved either through discussion or with a third reviewer.

### 2.5 Data Analysis and Presentation

Extracted data were presented in narrative format and in tables. For each cancer type, a group of three to four authors conducted quality checks of the extracted data. Data analysis involved synthesizing the extracted data to identify themes with consensus or divergence, with respect to LOT classification. The current paucity of evidence‐based performance metrics or validation studies that evaluate algorithms which identify initiation of LOT for locally advanced or metastatic disease in RWD precluded formal comparative evaluation of algorithm accuracy, validity, or generalizability.

### 2.6 Ethical Considerations

This study did not collect individual‐level data on any human subjects. It was exempted from review by the WIRB‐Copernicus Group Institutional Review Board because it does not meet the definition of human subject research as defined in 45 CFR 46.102 (e) [1].

## 3 Results

### 3.1 Summary of the Scoping Review

The initial search strategy identified 273 publication records. Of these, duplicates and studies published before 01/01/2014 were removed, leaving 120 (lung), 46 (breast) and 56 (colorectal) titles/abstracts. After screening, 28 (lung), 30 (breast) and 23 (colorectal) full‐text reports were assessed for study eligibility, and 25 studies overall were included in the final analysis (Figure 1) [7, 13, 14, 15, 16, 17, 18, 19, 20, 21, 22, 23, 24, 25, 26, 27, 28, 29, 30, 31, 32, 33, 34, 35, 36]. One of the included studies [22] was identified in all three cancer types, and another [7] was identified in both lung and colorectal cancer.

### 3.2 Data Sources of the Included Studies

All 25 studies were original research articles, although three of them were identified through a gray literature search [16, 22, 24]. There were 16 (64%) studies from North America [7, 13, 14, 15, 16, 17, 20, 22, 24, 25, 27, 28, 30, 32, 35, 36]; four (16%) from Europe [14, 17, 21, 33]; four (16%) from Asia [22, 26, 34, 36]; and one (4%) from Africa (Table 1) [31]. No studies from Latin America or the Gulf Cooperation Council (GCC) region met eligibility criteria. Eleven studies were published from 2014–2019 and 14 from 2020–2024. Several studies implemented RWD algorithms across multiple data sources. Overall, algorithms were implemented in EHR (18 [72%]), administrative healthcare claims (12 [48%]), cancer registries (7 [28%]), and chart reviews (4 [16%]).

**TABLE 1:** Characteristics of studies which have described approaches for identifying lines of therapy (LOTs) for non‐metastatic, relative to metastatic lung, breast, and colorectal cancer in real‐world data, 2014–2024.

First author, year	Real‐world data source(s), location	Included cancer types (histology, molecular, and stage)	Included systemic anticancer treatments (SACTs)	Approach for identifying completion of LOTs for non‐metastatic cancer	Approach for identifying initiation of LOTs for metastatic cancer	Approach for identifying transition from LOTs for non‐metastatic, to metastatic cancer	Any other approach for enumerating LOTs in cancer
*Lung cancer*							
Choi 2021	EHR, Claims, US	NSCLC; Stage IV		X	—	X	—
Cortellini 2021	EHR, Registry, Europe	Advanced NSCLC, PD‐L1 expression	Pembrolizumab, chemotherapy, others	X	—	X	—
Hess 2021	EHR, Claims, US	Advanced NSCLC; Stage IIIB or IV	Chemotherapy, targeted therapy, immunotherapy	X	—	X	—
Jeon 2021	EHR, Claims, S Korea	Histology, receptor status, and stage unspecified;	Chemotherapy	X	X	X	—
Korytowsky 2018	Claims, US	Advanced NSCLC	Not reported	X	—	X	—
Meng 2019	Claims, US	NSCLC; Stage IV	Anti‐PD‐1/PD‐L1, EGFR/ALK inhibitors, VEGF inhibitors, Platinum‐based chemotherapy, other	X	—	X	—
Meng 2021	EHR, Chart Review, US	NSCLC; Stage IV	Chemotherapy, EGFR inhibitors, immunotherapy	X	—	X	—
Sørup 2022	Registry, Denmark	Advanced NSCLC	Chemotherapy	X	—	X	—
*Breast cancer*							
Antoine 2023	EHR, France	Histology unspecified; HER2‐; Stage IV	Paclitaxel, bevacizumab	X	—	X	X
Cui 2021	EHR, US	Histology unspecified; HR+/HER2−; Stage 0–IV	CDK4 & 6 inhibitors, chemotherapy, endocrine therapy	X	—	X	—
DeMichele 2023	EHR, US	Ductal, lobular, mixed; HR+/HER2−; Stage IV	CDK4 & 6 inhibitor (palbociclib), aromatase inhibitors	X	—	X	X
Jeon 2021	EHR, Claims, S Korea	Histology, receptor status, and stage unspecified	Chemotherapy	X	X	X	—
Kish 2018	EHR, US	Ductal, lobular, unknown; HR+/HER2−; Stage IV	CDK4 & 6 inhibitor (palbociclib)	X	—	X	—
Liang 2015	Claims, US	Histology, and receptor status unspecified; Stage IV	Nab‐paclitaxel	X	—	X	—
Liu 2022	EHR, China	Histology unspecified; HR+/HER2−; Stage IV	Endocrine therapy, Chemotherapy	X	—	X	—
Merola 2022	EHR, US	Histology unspecified; HR+/HER2−; Stage IV	Palbociclib and fulvestrant, palbociclib and letrozole	X	—	X	X
Murthy 2014	Hospital database, US	Histology unspecified; HER2+; Stage I–IV	Trastuzumab‐based therapy	X	—	X	X
Schwartz 2018	Claims, Registry, US	Histology unspecified; Triple‐negative; Stage III/IV	Surgery + chemotherapy; Chemotherapy ± radiotherapy	X	—	X	—
Wang 2022	EHR, US	Histology unspecified; HR+/HER2‐; Stage IV	CDK4 & 6 inhibitor (abemaciclib), aromatase inhibitors	X	—	X	—
*Colorectal cancer*							
Abrams 2014	Registry, Intellidose, US	Stage IV	Chemotherapy without biologic agents, Bevacizumab + Chemotherapy, EGFR inhibitors (cetuximab, panitumumab) + chemotherapy	—	—	—	—
Bikov 2015	Claims, Registry, US	Stage IV	Chemotherapy, Bevacizumab, EGFR inhibitors (cetuximab, panitumumab), Non‐NCCN listed drugs	X	—	X	—
Dotan 2014	Claims, US	Stage IV	Anti‐EGFR, No Anti‐EGFR	X	—	X	—
Gall 2014	EHR, Registry, UK	Stage IV rectal cancer (i.e., synchronous liver metastases)	5‐FU/oxaliplatin based chemotherapy, Cetuximab, Bevacizumab	X	—	X	—
Hess 2021	EHR, Claims, US	Advanced Colorectal; Stage IIIB or IV	Chemotherapy, targeted therapy, immunotherapy	X	—	X	—
Jeon 2021	EHR, Claims, S Korea	Unspecified	Chemotherapy	X	X	X	—
Rais 2024	EHR, Registry, Chart Reviews, Morocco	Adenocarcinoma, mucinous; Stage IV, including with unresectable metastases	Bevacizumab + Chemotherapy (FOLFOX, XELOX, FOLFIRI, XELIRI)	X	—	X	—
Varol 2014	Chart Review, Turkey	Stage III, IV	Chemotherapy	X	—	—	X
Wonglhow 2023	EHR, Thailand	Stage IV, including recurrent metastases	Chemotherapy	—	—	—	X

Abbreviations: ALK, anaplastic lymphoma kinase; CDK, cyclin‐dependent kinase; EGFR, epidermal growth factor receptor; EHR, electronic health record that is, patients' digital medical records which are maintained by healthcare providers; FOLFIRI, (leucovorin calcium + fluorouracil + irinotecan hydrochloride); FOLFOX, (leucovorin calcium + fluorouracil + oxaliplatin); HER2, human epidermal growth factor receptor 2; HR, hormone receptor; ICD, International Classification of Diseases; LOT, line of therapy; NCCN, National Comprehensive Cancer Network; NSCLC, non‐small cell lung cancer; PD‐L1, Programmed Death‐Ligand 1; Registry refers to purpose‐built databases intended to collect uniform data on defined populations (e.g., patients with solid tumor cancers); RWD, real world data, including data from chart reviews, claims data, and EHR data. Chart reviews refer to manual assessments of patient's electronic health records for research; Claims data are requests for payment made by healthcare providers for specific, itemized health services rendered to health‐insurance covered patients. Claims are prepared for reimbursement purposes, and the data variables are different from medical charts; SACT, systemic anti‐cancer therapy; US, United States; VEGF, vascular endothelial growth factor; XELIRI, (capecitabine + irinotecan hydrochloride); XELOX, (capecitabine + oxaliplatin).

### 3.3 Algorithms for Identifying Initiation of a New LOT for Metastatic Lung, Breast, and Colorectal Cancer

Twenty‐four of 25 studies described an algorithm for determining initiation of a new LOT for metastatic lung (*n* = 7) [7, 16, 17, 24, 27, 28, 33], breast (*n* = 10) [14, 18, 19, 23, 25, 26, 29, 30, 32, 35] or colorectal (*n* = 8) [7, 13, 15, 20, 21, 31, 34, 36] cancer (Table 2). Jeon et al. described an algorithm for enumerating LOT without focusing on identifying the initiation of a new LOT for metastatic disease [22]. Most algorithms (*n* = 23) required an incident diagnosis code for metastatic cancer after a variable lookback period with no evidence of metastatic disease [7, 13, 14, 15, 16, 18, 19, 20, 21, 23, 24, 25, 26, 27, 28, 29, 30, 31, 32, 33, 34, 35, 36]. A new SACT after this incident diagnosis code was deemed the initiation of a new LOT for metastatic cancer (Table S3). Additional approaches for identifying initiation of a new LOT for metastatic cancer relied on the observation of a new SACT after other incident events which were potentially indicative of metastatic progression (new EHR or registry record of stage III/IV breast [23, 32] or colorectal [7] cancer). Six studies (24%) explored algorithms that considered the start of a new SACT after a sufficient treatment gap as initiation of a new LOT for metastatic disease (irrespective of incident metastatic diagnosis record) [7, 14, 23, 27, 28, 35]. For example, Hess et al. and Korytowsky et al. defined initiation of a new LOT as the observation of a SACT after a no‐treatment interval of 60, 90, 120, or 180 days [7, 24]. Wang et al. and Antoine et al. defined progression to a more advanced LOT as initiation of a new SACT after a specified treatment gap, without requiring an incident diagnosis of metastatic breast cancer [14, 35].

**TABLE 2:** Published RWD approaches for identifying the initiation of LOTs for metastatic cancer, or the transition point from LOTs for non‐metastatic, to LOTs for metastatic disease, among adults with lung, breast, or colorectal cancer, 2014 to 2024.

First author, year, location	Approaches for identifying completion of LOTs for non‐metastatic cancer	Approach for identifying initiation of LOTs for metastatic cancer	Approach for identifying transition from LOTs for non‐metastatic, to metastatic cancer
*Lung cancer*			
Choi 2021, US	N/A	The first SACT received after observing an incident diagnosis code for metastatic disease was deemed to be the initiation of LOTs for metastatic cancer. An incident diagnosis code for metastatic cancer was defined as no prior metastasis diagnosis in all available baseline in the study database before the earliest diagnosis code for metastatic cancer was observed.	N/A
Cortellini 2021, Europe	N/A	The earliest pembrolizumab treatment received after observing metastatic cancer was deemed to be the initiation of LOTs for metastatic cancer.	N/A
Hess 2021, US	N/A	The first SACT received after observing an incident diagnosis code for metastatic disease was deemed to be the initiation of LOTs for metastatic cancer. Four other RWD studies which used this approach were cited [37, 38, 39, 40]. Hess et al., 2021 further deemed that all SACTs received within the first 28‐days of initiating first‐line treatment for metastatic cancer are part of the first LOT.	A rule based on observing a treatment gap of 60 days between completion of a SACT and restarting the same SACT with an accompanying incident diagnosis of NSCLC was used to indicate transition to a new LOT. However, this study did not explicitly apply this concept to determining the transition point between the completion of last LOT for non‐metastatic disease, and initiation of the first LOT for metastatic disease.
Korytowsky 2018, US	N/A	The first SACT received after observing an incident diagnosis code for advanced disease was deemed to be the initiation of LOTs for advanced cancer. However, a treatment gap of 12 months was additionally required before the first SACT for advanced cancer, and this first SACT needed to have occurred within 6 months after the incident diagnosis of advanced disease for the SACT to be deemed as the initiation of LOT for advanced cancer.	N/A
Meng 2019, US		The first SACT received after observing an incident diagnosis code for metastatic disease was deemed to be the initiation of LOTs for metastatic cancer. Also, the introduction of a new SACT and/or the discontinuation of an existing one was mentioned as a potential signal of an intentional adjustment in the treatment strategy due to disease progression (or other clinical factors). Thus, this approach deduces that the first observation of a specific treatment approved for metastatic cancer resulting in a change in the treatment regimen could be applied to indicate initiation of LOT for metastatic disease.	Rules based on treatment gaps of 120 to 180 days between completion of last LOT and a new LOT were articulated. However, this study did not explicitly apply these rules to determine the transition point between the completion of last LOT for non‐metastatic disease for an individual in the study, and same individual's initiation of the first LOT for metastatic disease.
Meng 2021, US	N/A	The first SACT received after observing an incident diagnosis code for advanced disease was deemed to be the initiation of LOTs for advanced cancer	Rules based on treatment gaps of 120 to 180 days between completion of last LOT and a new LOT were articulated. However, this study did not explicitly apply these rules to determine the transition point between the completion of last LOT for non‐metastatic disease for an individual in the study, and same individual's initiation of the first LOT for metastatic disease.
Sørup 2022, Denmark	N/A	Metastatic cancer status was first confirmed using cancer registries. Once the date of metastatic cancer diagnosis was identified, the first SACT observed after this date was deemed to be the initiation of LOTs for the advanced cancer setting	N/A
*Breast cancer*			
Antoine 2023, France	N/A	The first SACT received after observing an incident diagnosis code for metastatic disease was deemed to be the initiation of LOTs for metastatic cancer. However, in addition, a clinically relevant “grace period” of 1 month before through 4 months (i.e., 120 days) after the confirmatory diagnosis of metastatic cancer was specified as the allowable period within which initiation of LOT for metastatic disease needed to have occurred.	N/A
Cui 2021, US	N/A	The first SACT received after observing an incident diagnosis code for metastatic disease was deemed to be the initiation of LOTs for metastatic cancer. However, initiation of LOT for metastatic disease needed to have been observed within 90 days after the confirmatory diagnosis date of metastatic disease, else the woman was excluded from the study.	N/A
DeMichele 2023, US	N/A	The first SACT received after observing an incident diagnosis of metastatic disease was deemed to be the initiation of LOTs for metastatic cancer.	N/A
Kish 2018, US	N/A	The first SACT observed following metastatic diagnosis was deemed the initiation of LOT for metastatic disease. Metastatic diagnosis date was defined as the earliest observed record of any of the following: American Joint Committee on Cancer (AJCC) stage IV, ICD‐9/10‐CM diagnosis code for metastatic disease, or M1 stage. However, if none of these events were recorded in the EHR, the date of the first treatment order for palbociclib was assigned as the metastatic diagnosis date (and thus, the date of initiation of LOT for metastatic disease).	N/A
Liang 2015, US	N/A	The first observed receipt of nab‐paclitaxel after diagnosis of metastatic cancer was deemed the initiation of LOT for metastatic disease.	N/A
Liu 2022, China	N/A	The first observed SACT (either chemotherapy or endocrine therapy) after diagnosis of metastatic cancer was deemed the initiation of LOT for metastatic disease.	N/A
Merola 2022, US	N/A	The first ordering record for a SACT combination (palbociclib and fulvestrant, relative to palbociclib and letrozole) observed following metastatic diagnosis was deemed the initiation of LOT for metastatic disease.	N/A
Murthy 2014, US	N/A	The first observed receipt of trastuzumab‐based therapy after diagnosis of metastatic HER2‐positive cancer was deemed the initiation of LOT for metastatic disease.	N/A
Schwartz 2018, US	N/A	The first SACT observed after diagnosis of stage III/IV disease was deemed the initiation of LOT for metastatic disease. All SACTs received within 15 days of this first SACT were included in the definition of first LOT for metastatic disease.	N/A
Wang 2022, US	N/A	The first SACT (either anastrozole or letrozole) observed after diagnosis of metastatic disease was deemed the initiation of LOT for metastatic disease if there was no evidence or history of receiving a previous SACT for advanced disease.	N/A
*Colorectal cancer*			
Abrams 2014, US	End of last adjuvant chemotherapy received for non‐metastatic disease, plus a 90‐day interval.	The first SACT (chemotherapy agents) observed after diagnosis of metastatic disease was deemed the initiation of LOT for metastatic disease. Additionally, this initial SACT or a substitute agent for metastatic cancer must have persisted for at least 28 days, else the study participant was excluded.	The transition point to LOTs for metastatic disease was defined based on observing the first SACT (chemotherapy agents) after diagnosis of metastatic disease, AND after at least 90 days had lapsed since the completion of adjuvant SACTs (chemotherapy) for non‐metastatic cancer. This algorithm further required that the initial LOT for metastatic cancer must have persisted for at least 28 days, else the record was ineligible.
Bikov 2015, US	N/A	The first SACT or SACT combination observed after diagnosis of metastatic disease, whether the SACT was listed by NCCN guidelines for colon cancer or not, was deemed the initiation of LOT for metastatic disease.	N/A
Dotan 2014, US	N/A	The first observed claim for a SACT after the first diagnosis code for metastatic disease was deemed to be the initiation of LOTs for metastatic cancer.	N/A
Gall 2014, UK	N/A	The first SACT (peri‐operative neoadjuvant chemotherapy) received after presenting with metastatic (liver metastases) rectal cancer at diagnosis was deemed the initiation of first LOT for metastatic cancer.	N/A
Hess 2021, US	N/A	The first SACT received after observing an incident diagnosis code for metastatic disease was deemed to be the initiation of LOTs for metastatic cancer. Four other RWD studies [37, 41, 42, 43] which used this approach were cited. Hess et al., 2021 further deemed that all SACTs received within the first 28‐days of initiating first‐line treatment for metastatic cancer are part of the first LOT.	A rule based on observing a treatment gap of 180 days between completion of a SACT and restarting the same SACT with an accompanying incident diagnosis of colorectal cancer was used to indicate transition to a new LOT. However, this study did not explicitly apply this concept to determining the transition point between the completion of last LOT for non‐metastatic disease, and initiation of the first LOT for metastatic disease.
Rais 2024, Morocco	N/A	The first observed SACT combination (bevacizumab in combination with standard chemotherapy) after a record of incident histopathologic diagnosis of metastatic disease and unresectable metastases	N/A
Varol 2014, Turkey	End of last adjuvant chemotherapy or radiotherapy received for non‐metastatic disease, plus at least 6 months interval free of adjuvant SACTs.	The first observed SACT (single agent chemotherapy cycle or combination) after an incident diagnosis code for metastatic disease was deemed to be the initiation of LOTs for metastatic cancer.	After completion of adjuvant chemotherapy or radiotherapy for non‐metastatic colorectal cancer, the transition to LOTs for metastatic disease was based on having a 6 or more months' adjuvant chemotherapy or radiotherapy‐free interval before an incident diagnosis of metastatic disease followed by incident treatment with a new SACT (irinotecan, oral 5‐FU derivatives) or SACT combination was observed.
Wonglhow 2023, Thailand	End of last adjuvant chemotherapy received for non‐metastatic disease, plus at least 6 months interval of disease‐free status.	The first observed chemotherapy cycle after an incident diagnosis code for metastatic or recurrent disease was deemed to be the initiation of LOTs for metastatic cancer.	After completion of adjuvant chemotherapy for non‐metastatic colorectal cancer, the transition to LOTs for advanced metastatic disease was characterized as observation of a new chemotherapy cycle (FOLFOX, FOLFIR, CAPOX or CAPIRI regimen) with an accompanying diagnosis of metastatic/recurrent disease. The new chemotherapy cycle must have occurred after a 6 or more months' disease‐free interval after the completion of prior adjuvant chemotherapy for non‐metastatic disease.

Abbreviations: 5FU, 5‐fluorouracil; CAPIRI, (capecitabine + irinotecan hydrochloride); CAPOX, (capecitabine + oxaliplatin); CM, clinical modification; EHR, electronic health record; FOLFIR, (leucovorin calcium + fluorouracil + irinotecan hydrochloride); FOLFOX, (leucovorin calcium + fluorouracil + oxaliplatin); HER2, human epidermal growth factor receptor 2; ICD, International Classification of Diseases; LOT, line of therapy; NCCN, National Comprehensive Cancer Network; NSCLC, non‐small cell lung cancer; RWD, real world data; SACT, systemic anti‐cancer therapy.

Four (16%) studies defined initiation of a new LOT for metastatic cancer as the observation of a specific SACT combination. Meng et al., 2019, Meng et al., 2021, and Hess et al. defined initiation of a new LOT for metastatic lung and colorectal cancer as the observation of a new SACT or a change in regimen [7, 27, 28]. Kish et al. classified patients as newly initiating LOT based solely on first palbociclib order if no diagnosis code for metastatic cancer, Stage IV, or M1 stage was recorded [23].

### 3.4 Algorithms for Identifying the Transition Point Between Completion of LOTs for Non‐Metastatic Disease and Initiation of LOTs for Metastatic Disease

Three colorectal cancer studies described methods for identifying the transition from completion of adjuvant LOTs for non‐metastatic disease to initiation of LOTs for metastatic disease (Table 2) [13, 34, 36]. All three algorithms specified an interval after the end of adjuvant treatment for non‐metastatic cancer (without observed adjuvant treatments or disease progression) before a new diagnosis of metastatic disease and accompanying re‐initiation of LOTs. Two studies [34, 36] required this interval to be ≥ 6 months, while Abrams et al. required a shorter interval of ≥ 3 months (Table S3) [13]. This study further required that the first LOT after metastatic colorectal cancer diagnosis persist for ≥ 28 days to be considered first‐line [13].

### 3.5 Validation of Described RWD Algorithms

Among included studies, three (12%) validated their algorithm(s) [7, 27, 33]. Hess et al. validated EHR and claims algorithms by visually comparing SACT regimens and LOT sequences captured by their algorithms with those expected for first‐line metastatic cancer treatment per guidelines [7]. Practicing oncologists were invited to assess when LOT sequences from the study algorithms best resembled treatment patterns in clinical practice (face validity); however, validation results were not presented in the paper [7]. Sørup et al. and Meng validated algorithms using a reviewer‐blinded manual chart or EHR review [27, 33]. In Sørup et al., agreement with the reference standard (LOT changes from medical records) was higher for the algorithm requiring a specific SACT combination than the algorithm requiring a 45‐day treatment gap duration between SACTs (91% vs. 68%) [33]. Meng 2021 showed that the distribution of first‐line LOT counts defined as receipt of SACT after incident diagnosis code for metastatic lung cancer was similar (55.1% vs. 58.0%) between training and test cohorts evaluated against reference chart review [27].

## 4 Discussion

Our scoping review summarizes algorithms for identifying LOTs for metastatic lung, breast, or colorectal cancer in RWD. Incident metastatic diagnosis was commonly required to classify new LOTs for metastatic disease. However, significant heterogeneity exists in treatment gap durations and the events signaling onset of or transition to metastasis.

EHRs were predominantly used to identify LOT. Most data emanated from North America, with little to no representation from Africa, Latin America, or the Gulf Cooperation Council. Regional disparities in access to oncology care and availability of EHR RWD may explain these findings [44, 45]. Differences in coding systems and incomplete capture of cancer staging information in certain RWD assets may preclude or confound LOT identification, and thereby, SACT effectiveness or safety research in certain regions. These issues have important implications for the equity and generalizability of real‐world oncology findings. RWD from under‐represented regions, socioeconomic, racial, and ethnic groups would enhance representation in future clinical cancer research and could be used to inform current LOT algorithms [46]. EHR data and claims assets are often challenged with missing data, coding errors, and lack of information on treatment intent. Systematic efforts to reduce these limitations, including data linkages (across claims, EHR, and registry sources) and the application of artificial intelligence to extract additional EHR information could enrich current data sources for improved performance and portability of LOT algorithms.

A key finding from our scoping review is the heterogeneity of events specified in RWD algorithms to denote the onset of metastatic cancer. Most studies described algorithms which relied on the observation of an incident International Classification of Diseases, Ninth or Tenth Revision, Clinical Modification (ICD‐9/10‐CM) diagnosis code for metastatic cancer. Others explored events which were potentially indicative of metastatic progression (new EHR or registry record of stage III/IV breast [23, 32] or colorectal cancer) [7]. This variability likely reflects differences in study objectives and data structure, including availability of clinical variables across RWD sources, and may limit generalizability and portability of algorithms across different RWD types. Given evidence of low positive predictive values (14% to 47%) when using secondary malignancy codes to identify metastatic lung, breast or colorectal cancer in claims data, researchers should explore using multiple criteria to determine LOT. Using a single criterion could potentially increase bias [47]. However, using multiple criteria could decrease sample sizes, as not all patients may have the required data elements. The optimal approach, therefore, would be to carefully select a minimal set of criteria that balances bias minimization and maintaining a sufficient sample size.

The selection of an appropriate treatment gap definition for identifying new LOTs should be informed by the drug's pharmacokinetic properties (particularly half‐life), expert clinical opinion, and sensitivity analyses. Common treatment gap definitions of 60, 90, 120, and 180 days, depending on cancer type, are widely accepted in the pharmacoepidemiologic field. Future researchers may consider a single, drug‐specific gap definition and include sensitivity analyses with alternative gap definitions.

In comparing specific algorithmic components, wide heterogeneity and low inter‐study comparability were observed between algorithms used to identify first LOT in lung cancer studies. This may indicate that study‐specific decisions impact algorithm designs. For breast cancer studies, greater homogeneity was observed in the identification of the first LOT for metastatic cancer. Among colorectal cancer studies, there was greater heterogeneity in the type of criteria used to indicate metastasis onset and in the length of treatment gaps to indicate transition to LOT for metastatic disease. Establishing a consensus algorithm which can be implemented with validity across studies may remain a challenge due to variability in clinical guidelines by geography and targeted treatments. Minimum criteria for identifying the initiation of LOT for metastatic disease across all three cancer types should include observation of a new metastatic cancer diagnosis and a minimum 180 day lookback period which confirms no prior treatment with SACTs indicated for metastatic cancer.

Querying for baseline completion of LOTs for non‐metastatic cancer before flagging initiation of new LOTs for metastatic disease is recommended where possible (Figure 2). Such algorithms help to distinguish initiators of new LOTs for metastatic cancer who had benefitted from earlier diagnosis and adjuvant treatment from those presenting with metastases at the point of cancer diagnosis (late presentation). Prognosis may differ between these two groups. Variability in gap periods used to define the transition from completion of non‐metastatic LOTs to initiation of metastatic LOTs may introduce potential misclassification bias. Shorter intervals, like the 3 months used by Abrams et al., may cause false‐positive misclassification by misidentifying ongoing adjuvant therapy or treatment breaks as metastatic LOT initiation [13]. Longer intervals, such as the 6 months applied by Varol et al., risk false‐negative misclassification by delaying or missing metastatic LOT onset, underestimating treatment transitions and reducing temporal accuracy [34].

Opportunities abound to improve future RWD approaches for identifying initiation of LOTs for metastatic cancer. Thompson proposed a framework for determining new LOTs based on clinician‐recorded disease progression dates in the UK [5]. Additionally, Saini and Twelves proposed a framework for enumerating LOTs for solid cancers [4]. These frameworks require standardized LOT reporting in RWD, including classifying the intent for LOTs as either curative or palliative [4]. Only three studies described formal validation procedures, and only two reported performance metrics. Internal validation of algorithms using blinded chart/EHR review, expert consensus or performance metrics, and external validation in diverse populations and healthcare systems are critical in future research to advance reproducibility and generalizability [41]. Our findings demonstrate the need for interdisciplinary consensus‐building among oncologists, epidemiologists, and data scientists. Standardization of definitions for LOT progression and implementation of best practices, including algorithm validation and granularity in reporting algorithms and assumptions used, would enable peer review and replication in RWD oncology research.

Our scoping review offers a timely and methodologically robust synthesis of RWD algorithms for identifying LOTs in metastatic lung, breast, and colorectal cancers. Adherence to JBI and PRISMA‐ScR standards, with protocol registration on OSF, ensures transparency, reproducibility, and a comprehensive, structured overview of 25 studies across diverse regions and data sources. Our systematic categorization of initiation criteria, lookback windows, and transition markers enhances clarity in a field marked by heterogeneity. This template for identifying first LOT for metastatic solid tumors may improve inclusion/exclusion criteria for clinical trials and in outcomes research, enhance identification of time zero and improve outcome comparisons [42, 43]. Precise enumeration of first and subsequent metastatic LOTs in RWE will inform therapeutic decision making and health services planning by cancer stage in the clinical setting. As the first known review to empirically map LOT algorithms across these three cancer types in RWD, our study provides a foundational step toward future methodological standardization and validation efforts.

Nevertheless, this scoping review presents some limitations that warrant consideration. The review focused exclusively on SACTs, excluding radiotherapy, surgery, and other modalities commonly used in metastatic cancer care, which may limit the comprehensiveness of our insights on treatment trajectories. Although we followed a rigorous methodological framework, including dual‐review screening and data extraction, lack of standardized performance metrics, limited reporting of validation outcomes, and heterogeneity of available data prevented evaluation of the accuracy, validity, or generalizability of included studies. While our search strategy was comprehensive, restriction to English‐language publications may have introduced selection bias. We recommend further research in other languages, other treatment modalities (surgery, radiotherapy), hematological malignancies, and adoption of open‐source code/tools for algorithm transparency and uptake.

## 5 Conclusions

Although most algorithms used to define LOT in RWD identified a new SACT following metastatic diagnosis, there is significant heterogeneity in key factors. These include definitions of metastatic onset, required treatment gap durations after completion of non‐metastatic LOTs, and consistency in querying for completion of non‐metastatic LOTs before classifying new SACTs as initiation of LOTs for metastatic cancer. Although some differences in LOT algorithms are expected due to the nature of RWD sources, our review highlights actionable opportunities to enhance methodological rigor and comparability. Investigators are encouraged to validate and transparently publish the algorithms and assumptions they use, enabling peer review and replication. Without such transparency and reproducibility, cross‐study comparisons and international benchmarking efforts will remain methodologically constrained. Interdisciplinary groups comprised of oncologists, data scientists, and pharmacoepidemiologists should collaborate to develop consensus‐driven definitions and frameworks for LOT classification. Overall, more accurate algorithmic identification of LOT may improve treatment outcome comparisons, therapeutic decision‐making, and health services planning across global oncology settings.

## Author Contributions

The lead author (O.O.) contributed to the study design and drafted the manuscript. All authors made substantial contributions to the interpretation of the data and results, reviewed, provided critical revisions, and approved the final manuscript.

## Funding

This work was supported by Carelon Research and the International Society for Pharmacoepidemiology.

## Ethics Statement

This study was deemed exempt from ethics review by the WIRB‐Copernicus Group Institutional Review Board.

## Conflicts of Interest

O.O. is an employee of Carelon Research and works on contract research for life sciences companies, payers, and government agencies. D.B. is an employee of Takeda. I.M.S. is an employee of Merck and Co. Inc., Rahway, NJ, USA. M.S. is an employee of Bayer Pharmaceuticals. S.H.M. Employment: Merck Healthcare KGaA, Darmstadt, Germany; stock and other ownership interests: Bayer AG. The remaining authors declare no conflicts of interest.

## Supporting information


**Appendix S1:** Search Strategies for Lung, Breast, and Colorectal Cancer.
**Table S1:** Data Extraction Tool.
**Table S2:** Post‐Protocol Modifications Made to Data Extraction Tool.

## Data Availability

All data generated or analyzed during this study are included in this published article.
