# Supplementary material for: Identifying the Initiation of a New Line of Therapy for Metastatic Lung, Breast, and Colorectal Cancer in Real‐World Data: A Scoping Review
Source: Pharmacoepidemiol Drug Saf. 2026 Apr 27;35:e70370. doi: 10.1002/pds.70370 (PMC13121922; doi:10.1002/pds.70370)
Supplement: Supplementary file 1 — Appendix S1: Search Strategies for Lung, Breast, and Colorectal Cancer. Table S1: Data Extraction Tool. Table S2: Post‐Protocol Modifications Made to Data Extraction Tool. [file PDS-35-e70370-s001.docx]

**Appendix 1. Search Strategies for Lung, Breast, and Colorectal Cancer**

PubMED Search: Lung* AND (“claims data” OR “administrative data” OR “claims database” OR “administrative database” OR “observational” OR “real world” OR “secondary data” OR “health record” OR “medical record” OR chart OR registry OR registries OR retrospective) AND (“line of treatment” OR “line treatment” OR “treatment line” OR “therapy sequence” OR “sequence of therapy” OR “Therapy line” OR “line of therapy” OR “line therapy” OR “line regimen”) AND (Algorithm OR rules OR rule OR approach OR framework OR “machine learning”) AND (Cancer OR neoplasm OR neoplasia OR oncology)

Breast* AND (“claims data” OR “administrative data” OR “claims database” OR “administrative database” OR “observational” OR “real world” OR “secondary data” OR “health record” OR “medical record” OR chart OR registry OR registries OR retrospective) AND (“line of treatment” OR “line treatment” OR “treatment line” OR “therapy sequence” OR “sequence of therapy” OR “Therapy line” OR “line of therapy” OR “line therapy” OR “line regimen”) AND (Algorithm OR rules OR rule OR approach OR framework OR “machine learning”) AND (Cancer OR neoplasm OR neoplasia OR oncology)

(Colon* OR Colorectal*) AND (“claims data” OR “administrative data” OR “claims database” OR “administrative database” OR “observational” OR “real world” OR “secondary data” OR “health record” OR “medical record” OR chart OR registry OR registries OR retrospective) AND (“line of treatment” OR “line treatment” OR “treatment line” OR “therapy sequence” OR “sequence of therapy” OR “Therapy line” OR “line of therapy” OR “line therapy” OR “line regimen”) AND (Algorithm OR rules OR rule OR approach OR framework OR “machine learning”) AND (Cancer OR neoplasm OR neoplasia OR oncology)

**Table 1. Data Extraction Tool**

| **Parameters** | **Findings** |
| --- | --- |
| Author(s), publication year |  |
| Country, region of databases used |  |
| Real-world data types assessed |  |
| Electronic Medical/Health Record |  |
| Administrative Claims Database |  |
| Registry, including type of registry |  |
| Paper Medical Charts |  |
| Other RWD type |  |
| Setting from which LOT data is obtained |  |
| Routine healthcare setting |  |
| Surveys of clinicians, researchers, expert opinion |  |
| Study Design |  |
| Population (Age range, sex, race/ethnicity, sample size, cancer types, subtypes, cancer stage) |  |
| Age group |  |
| Sex |  |
| Race/ethnicity |  |
| Cancer subtypes assessed |  |
| Cancer stages assessed |  |
| Sample size of population assessed |  |
| Cancer therapies (SACTs) assessed |  |
| Does article describe any approach for identifying the transition point between LOTs used to treat non-metastatic lung, breast, or colorectal cancer; relative to LOTs used for metastatic disease? |  |
| Does article describe any approach for determining the completion of LOTs used with curative intent for non-metastatic lung, breast, or colorectal cancer? |  |
| Does article describe any approach for determining the commencement of LOTs used (with or without palliative intent) to treat metastatic lung, breast, or colorectal cancer? |  |
| Description of proposed approaches, rules, or frameworks for classifying LOT in the article. |  |
| Proposed algorithms for classifying LOT |  |
| Key variables used to define algorithm: |  |
| Disease progression? |  |
| Treatment response? |  |
| Treatment duration? |  |
| Specific treatment regimens? |  |
| Addition/removal of treatments? |  |
| Gap periods without treatment? |  |
| Comorbid diagnoses or procedures? |  |
| Place of service where LOT is received? |  |
| Consideration of maintenance therapy? |  |
| Others? |  |
| Does algorithm include programming code for identifying or classifying LOT in RWD? |  |
| Any best practices for classifying LOT recommended? |  |
| Is the approach for classifying LOT validated against a gold-standard where LOT and its treatment intent is known?  Note: Validated studies require reporting of evaluation metrics e.g. positive predictive value, negative predictive value, sensitivity, or specificity |  |
| Was performance of the LOT algorithm evaluated in any other way, apart from validation? |  |

**Table 2. Post-Protocol Modifications Made to Data Extraction Tool**

| **Parameters** | **Modifications** |
| --- | --- |
| Study Design | Extraction of study design information was dropped during implementation of the scoping review for relative lack of relevance to the research question |
| Does article describe any approach for determining the completion of LOTs used with curative intent for non-metastatic lung, breast, or colorectal cancer? | Extraction of data on this parameter was dropped during implementation of the scoping review as it was already captured when assessing approaches for the transition point between LOTs used to treat non-metastatic, relative to metastatic cancer |
